# Short-term dynamics of serum uric acid and its influencing factors in patients with obesity after laparoscopic sleeve gastrectomy

**DOI:** 10.1186/s12893-025-03437-z

**Published:** 2025-12-23

**Authors:** Haoyu Feng, Jingfeng Gu, Jian Zhang, Guiqi Wang

**Affiliations:** Department of Bariatric and Metabolic Surgery, The First Hospital of Hebei Medical University, No. 89 Donggang Road, Shijiazhuang, Hebei Province 050000 China

**Keywords:** Hyperuricemia, Laparoscopic sleeve gastrectomy

## Abstract

**Background:**

The pathophysiological association between obesity and hyperuricemia (HUA) is well-established. Metabolic and bariatric surgery (MBS) has been shown to effectively manage severe obesity and lead to sustained reductions in serum uric acid (SUA) over the long term; however, the factors modulating short-term fluctuations in SUA (i.e., within 6 months postoperatively) and their underlying mechanisms remain poorly elucidated.

**Methods:**

We performed a retrospective analysis of data from 184 patients with obesity who underwent laparoscopic sleeve gastrectomy (LSG). Clinical data were retrieved at baseline, on postoperative day 1, and at 1, 3, and 6 months postoperatively. Per established guidelines, patients were stratified into a normal SUA (NUA) group (n = 74) and an elevated SUA (EUA) group (*n* = 110).

**Results:**

Age, baseline estimated glomerular filtration rate (eGFR₀), baseline SUA (SUA₀), 1-month postoperative change in eGFR (Δ₁ₘ-eGFR), and 1-month postoperative change in BMI (Δ₁ₘ-BMI) were independent predictors of 1-month postoperative change in SUA (Δ₁ₘ-SUA).

Age, sex, SUA₀, baseline triglyceride-glucose index (TyG₀), eGFR₀, 6-month postoperative change in eGFR (Δ₆ₘ-eGFR), 6-month postoperative change in total protein (Δ₆ₘ-TP), and 6-month postoperative change in TyG (Δ₆ₘ-TyG) were independent predictors of 6-month postoperative SUA (SUA₆ₘ).

Process-mediated mediation analysis revealed that the effect of baseline BMI (BMI₀) on Δ₁ₘ-SUA was fully mediated by Δ₁ₘ-BMI; the effect of eGFR₀ on Δ₁ₘ-SUA was partially mediated by Δ₁ₘ-eGFR, while the effect of eGFR₀ on SUA₆ₘ was fully mediated by Δ₆ₘ-eGFR.

In the EUA group, 3-month postoperative SUA (SUA₃ₘ) was significantly lower than the baseline value. In the NUA group, SUA at 1, 3, and 6 months postoperatively (SUA₁ₘ/SUA₃ₘ/SUA₆ₘ) remained significantly higher than the baseline value, with a more pronounced increase in Δ₆ₘ-eGFR noted in males compared to females.

**Conclusion:**

Δ₁ₘ-BMI is a key determinant of Δ₁ₘ-SUA. Patients with Δ₁ₘ-BMI ≥ 4.25 kg/m^2^ warrant weekly SUA monitoring during the first month postoperatively. Prompt clinical intervention is necessary when SUA exceeds 535.5 μmol/L, especially in patients with a history of gout. For patients with baseline impaired renal function (eGFR: 60–89 mL/min/1.73m^2^), preoperative optimization of renal reserve is recommended; those with renal hyperfiltration (eGFR > 125 mL/min/1.73m^2^) require intensified postoperative monitoring of eGFR and SUA. Males in the NUA group are at an increased risk of SUA elevation at 6 months postoperatively. Furthermore, age and SUA₀ are independent predictors of Δ₁ₘ-SUA, while age, sex, SUA₀, TyG₀, Δ₆ₘ-TP, and Δ₆ₘ-TyG are independent predictors of SUA₆ₘ.

**Supplementary Information:**

The online version contains supplementary material available at 10.1186/s12893-025-03437-z.

## Introduction

According to projections from the *World Obesity Atlas 2025* [1], over 1.1 billion people worldwide will be living with obesity by 2030. Obesity is not only a major risk factor for type 2 diabetes, cardiovascular disease, and malignant tumors but also closely linked to the development of HUA [2–4]. Furthermore, persistent HUA further elevates the risk of gout, cardiovascular events, and chronic kidney disease [5].

Current first-line medications for HUA and gout in China are associated with multiple adverse effects, which limits their widespread clinical use [6]. MBS not only achieves long-term stable weight loss but also significantly improves SUA metabolism [7, 8]. However, existing studies [9, 10] indicate that in the early postoperative period (especially within the first month), patients experience marked fluctuations in SUA levels—accompanied by a parallel increase in the risk of acute gout flares. Currently, there is a paucity of systematic studies on the factors modulating short-term (particularly within 1 month) SUA fluctuations in patients with obesity undergoing LSG. Furthermore, the first six months after MBS constitute a critical period of rapid weight loss and serve as a key milestone for efficacy evaluation; yet the patterns of SUA metabolic alterations during this period remain incompletely characterized.

Given this research gap, this retrospective analysis was designed to address two core objectives: First, it focuses on the early postoperative period (within 1 month) to identify factors modulating short-term SUA fluctuations, with the goal of guiding targeted perioperative monitoring. Second, it examines the critical 6-month postoperative time point, systematically analyzing factors associated with SUA levels at this stage to offer clinical insights that inform the optimization of long-term metabolic management strategies.

## Methods

This retrospective observational study of patients with obesity who underwent MBS at our institution (2022–2024) involved no therapeutic interventions, with data solely retrieved from our institutional medical record system in July 2025.

### Patient selection

Inclusion criteria were: (1) Patients undergoing their first MBS via the LSG approach; (2) age between 16 and 65 years; (3) complete clinical data were available. Exclusion criteria were: (1) use of urate-lowering medications within 1 month prior to surgery or during follow-up; (2) development of major postoperative complications. Based on these criteria, we retrieved clinical data from 184 patients with obesity who underwent LSG at our hospital between 2022 and 2024. SUA levels were defined per the Chinese Guidelines for Diagnosis and Treatment of Hyperuricemia and Gout, with thresholds set at > 420 μmol/L for males and > 357 μmol/L for females. Patients were stratified into the NUA group (*n* = 74) and the EUA group (*n* = 110).

### Measurement

Case data were retrospectively retrieved at baseline, on postoperative day 1 (POD 1), and at 1, 3, and 6 months postoperatively. Retrieved indicators included height (cm), weight (kg), body mass index (BMI, kg/m^2^), waist circumference (WC, cm), SUA (μmol/L), serum creatinine (SCr, μmol/L), total protein (TP, g/L), triglyceride-glucose index (TyG), fasting blood glucose (FBG, mmol/L), triglycerides (TG, mmol/L), total cholesterol (TC, mmol/L), high-density lipoprotein cholesterol (HDL-C, mmol/L), low-density lipoprotein cholesterol (LDL-C, mmol/L), Chinese visceral adiposity index (CVAI), alanine aminotransferase (ALT, U/L), aspartate aminotransferase (AST, U/L), and apolipoprotein A1 (Apo A1, g/L). Given the limited accuracy of measurements during early postoperative recovery, patient weight, BMI, and WC were not recorded on POD 1.

Key derived variables were calculated using the following formulas:For patients aged > 18 years: The CKD-EPI Eq [11]. was applied: eGFR = 141 × min (Scr(mg/dL)/κ,1) ^−α^ × max (Scr(mg/dL)/κ,1) ^−1.209^ × 0.993^Age^ × 1.018[if female] × 1.159[if black] (κ = 0.7 for females, 0.9 for males; α = −0.329 for females, −0.411for males); For adolescent patients aged 16–17 years: The Schwartz Eq [12]. was used: eGFR = 0.55 × Height (cm)/SCr (mg/dL).

Excess Weight Loss Percentage (EWL%): [(Baseline BMI – Follow-up BMI)/(Baseline BMI – 25)] × 100% (with an ideal BMI set at 25 kg/m^2^).

Total Weight Loss Percentage (TWL%): [(Baseline Weight – Follow-up Weight)/Baseline Weight] × 100%

Triglyceride-Glucose Index (TyG): ln [TG (mg/dL) × FBG (mg/dL)/2].

Chinese Visceral Adiposity Index (CVAI):Male:−267.93 + 0.68 × Age + 0.03 × BMI (kg/m^2^) + 4.00 × WC(cm) + 22.00 × lg(TG, mmol/L) −16.32 × HDL-C(mmol/L); Female: 187.32 + 1.71 × Age + 4.32 × BMI + 1.12 × WC + 39.76 × lg(TG) −11.66 × HDL-C.

### Surgical procedure

All LSG procedures were performed laparoscopically by two experienced surgeons at our center. The standard surgical workflow for LSG was as follows: A standard three-port technique was employed. Starting 4 cm proximal to the pylorus, the greater curvature of the stomach was dissected upward along the inner margin of the gastroepiploic vascular arcade using an ultrasonic scalpel, extending to the angle of His. The posterior gastric wall was then mobilized to achieve complete freeing of the greater curvature. An assistant inserted a 36-Fr bougie transorally; a disposable laparoscopic linear cutting stapler was subsequently positioned, and vertical gastrectomy was performed 1 cm lateral to the bougie—starting 4 cm proximal to the pylorus and extending upward along the greater curvature to the gastric fundus—to resect the greater curvature gastric tissue. The omentum and the staple line on the greater curvature were continuously sutured with 3–0 absorbable sutures.

### Postoperative management and follow-up

On POD 1, patients were encouraged to engage in moderate activity and consume 600 mL of fluid. On POD 2, patients underwent a gastrointestinal contrast study. In the absence of complications or adverse events on POD 3, patients were eligible for discharge. Following discharge, attending physicians and registered dietitians collaborated to provide patients with comprehensive guidance, including dietary counseling (recommending 60–80 g of whey protein daily within 6 months postoperatively and guiding the transition of postoperative diet), physical activity, and nutritional supplementation.

### Statistical analysis

Statistical analyses were performed using SPSS version 27.0. Categorical variables are presented as frequencies (n, %). The normality of continuous variables was evaluated via the Kolmogorov–Smirnov test: normally distributed continuous variables are expressed as mean ± standard deviation (SD), while non-normally distributed variables are reported as median (interquartile range). Chi-square tests were utilized for comparing categorical variables. For normally distributed continuous variables, independent samples t-tests were conducted, with Pearson’s correlation coefficient (r) used to assess associations; for non-normally distributed continuous variables, Mann–Whitney U tests were employed, with Spearman’s correlation coefficient (ρ) for evaluating associations. Mediation analyses were performed using the PROCESS macro for SPSS. Multiple linear regression analysis was used to identify independent influencing factors. Repeated measures analysis of variance was applied to compare preoperative and postoperative SUA levels. A two-tailed p-value < 0.05 was considered statistically significant.

## Results

Initially, a total of 1988 patients with obesity who underwent MBS at our institution between 2022 and 2024 were identified as the starting cohort. A stepwise exclusion process was performed to refine the study population, as outlined below:

First, 2 patients were excluded because their ages were outside the 16–65-year range, leaving a final sample size of 1986. Subsequently, 36 patients were excluded for undergoing non-LSG MBS or revision surgery, leaving 1950 individuals eligible for further assessment. Additionally, 85 patients were excluded because they used urate-lowering medications either 1 month preoperatively or during the follow-up period, reducing the cohort to 1865. Finally, 1681 patients were excluded due to incomplete baseline data or postoperative follow-up data.

**Fig. 1:**
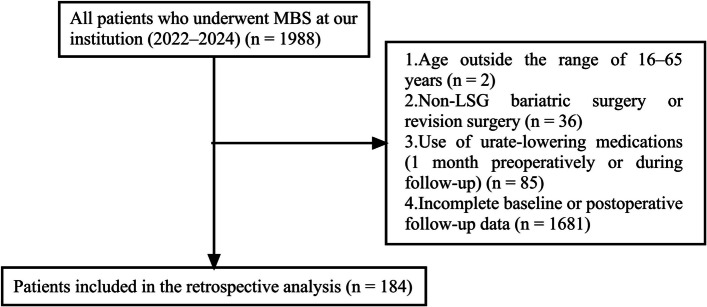
Flow diagram of the retrospective analysis

After application of all exclusion criteria, 184 patients were ultimately included in the retrospective analysis as the final study cohort. The detailed patient screening and enrollment process is illustrated in Fig. 1.

Baseline characteristics of the EUA and NUA groups are summarized in Table 1. Statistically significant differences between the two groups were observed in age, sex, SCr, TG, HDL-C, and Apo A_1_. Given the limited sample size of each group when analyzed independently—which may reduce statistical power—the two cohorts were combined for comprehensive statistical analyses to evaluate the impact of LSG on SUA levels in the overall study population.

**Table 1:** Baseline Characteristics of the EUA and NUA Groups

Variables	EUA	NUA	*χ2/t/Z*	*P"*
Age	32.28 ± 8.95	36.31 ± 7.48	−3.31	0.001**
Sex (Female%)	63(57.27)	54(72.97)	4.71	0.030*
Weight (kg)	118.12 ± 24.59	110.9 ± 26.41	1.9	0.059
BMI (kg/m^2^)	41.63 ± 6.87	40.07 ± 8.18	1.4	0.163
WC (cm)	124.33 ± 13.69	121.24 ± 15.25	1.43	0.154
SUA(μmol/L)	486.22 ± 80.39	317.71 ± 55.84	16.78	< 0.001***
SCr(μmol/L)	61.7 ± 14.93	54.45 ± 11.94	3.41	0.001**
eGFR(mL/min/1.73m^2^)	118.63 ± 13.1	119.27 ± 2.7	−0.34	0.736
TP(g/L)	74.33 ± 4.62	73.04 ± 4.62	1.86	0.064
TyG	9.14 ± 0.76	9.07 ± 0.86	0.55	0.583
FBG (mmol/L)	5.51(4.63, 6.86)	5.48(4.8, 7.59)	−0.71	0.479
TG (mmol/L)	1.8(1.37, 2.64)	1.48(1.16, 2.51)	−2.21	0.027*
TC (mmol/L)	5.35 ± 1.17	5.41 ± 1.06	−0.37	0.71
HDL-C(mmol/L)	1.17 ± 0.23	1.31 ± 0.29	−3.5	0.001**
LDL-C(mmol/L)	3.32 ± 0.79	3.32 ± 0.83	0.057	0.954
CVAI	212.47 ± 64.11	195.36 ± 66.76	1.75	0.082
Apo A_1_(g/L)	1.39 ± 0.26	1.48 ± 0.24	−2.43	0.016*
ALT(U/L)	38.3(23.65, 59.25)	36.35(18.7,64.1)	−0.77	0.443

### Factors Influencing Δ₁ₘ-SUA After LSG

**Fig. 2:**
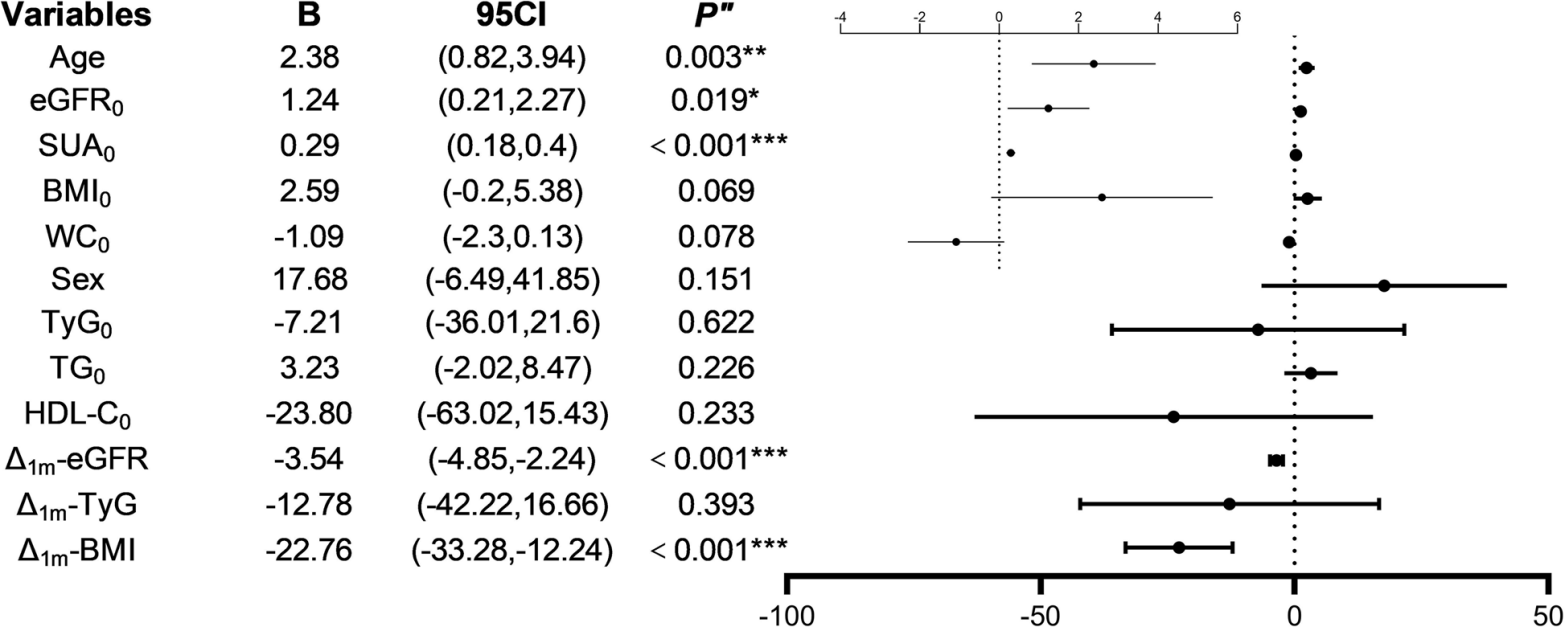
Forest plot of Δ_1m_-SUA independent predictors

**Fig. 3:**
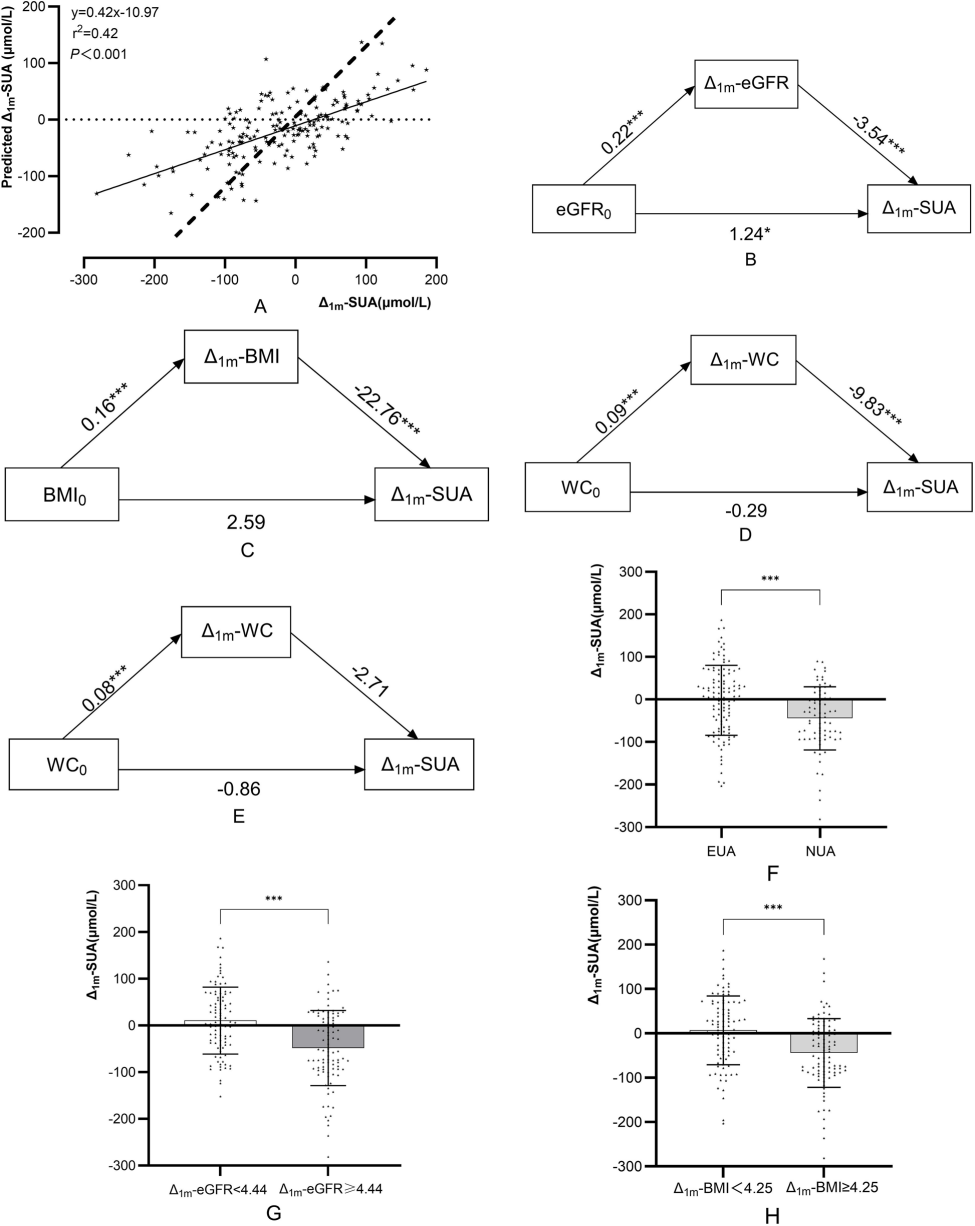
Multiple linear regression model fit for Δ_1m_-SUA (**A** The dashed line represents a slope of 1); Mediation effects of Δ_1m_-eGFR (**B**) and Δ_1m_-BMI (**C**); Mediation effects of Δ_1m_-WC without (**D**) and with (**E**) TWL_1_ adjustment; Stratified comparisons of Δ_1m_-SUA by SUA_0_ (**F**), Δ_1m_-eGFR levels (**G**), and Δ_1m_-BMI (**H**)

Pearson or Spearman correlation analysis was utilized to identify factors correlated with Δ₁ₘ-SUA. Considering sample size constraints, variables with significant multicollinearity were excluded, with adjustment for confounding factors including age, sex, baseline triglycerides (TG₀), and baseline high-density lipoprotein cholesterol (HDL-C₀). The final multiple linear regression model demonstrated (Fig. 2) that age, eGFR₀, SUA₀, Δ₁ₘ-eGFR, and Δ₁ₘ-BMI were independent predictors of Δ₁ₘ-SUA. The model explained 42% of the variance in Δ₁ₘ-SUA (R^2^ = 0.42; slope = 0.42, Fig. 3A).

### Mediation analysis of Δ₁ₘ-SUA

eGFR₀ was positively correlated with Δ₁ₘ-eGFR (r = 0.28, *p* < 0.001), whereas Δ₁ₘ-eGFR was negatively correlated with Δ₁ₘ-SUA (r = −0.43, *p* < 0.001). Similarly, baseline waist circumference (WC₀) was positively correlated with 1-month postoperative change in WC (Δ₁ₘ-WC, r = 0.58, *p* < 0.001), and Δ₁ₘ-WC was negatively correlated with Δ₁ₘ-SUA (r = −0.28, *p* < 0.001). BMI₀ was positively correlated with Δ₁ₘ-BMI (r = 0.74, *p* < 0.001), whereas Δ₁ₘ-BMI was negatively correlated with Δ₁ₘ-SUA (r = −0.31, *p* < 0.001). These results demonstrate direct correlations among the aforementioned variables; however, it remains unclear whether Δ₁ₘ-eGFR, Δ₁ₘ-WC, and Δ₁ₘ-BMI independently mediate the effects of eGFR₀, WC₀, and BMI₀ on Δ₁ₘ-SUA.

To validate the aforementioned mediating pathways, a simple mediation model was constructed using the PROCESS macro (developed by Hayes). Following standard guidelines proposed by Fritz & Mackinnon (2007) [13], we preset moderate effect sizes (path α = 0.39 for X → M and path β = 0.39 for M → Y), a significance level of 0.05, and a target power of 0.80. This yields a minimum effective sample size of 71 cases required to detect mediation effects. With a total sample size of 184 in the current study, this requirement was satisfied.

The 95% confidence interval for the indirect effect was first estimated via 5,000 percentile bootstrap samples. To test the stability of this estimate, the analysis was repeated with 10,000 samples. The results were virtually identical, indicating that the estimates had converged and that the findings are robust. An interval excluding zero denotes a significant indirect effect. Additionally, a joint significance test was utilized, requiring both path α (X → M) and path β (M → Y) coefficients to reach statistical significance (*p* < 0.05). If both criteria were satisfied, the mediation effect was considered statistically significant. To ensure consistency in statistical modeling, the strategy for incorporating confounders in the mediation analysis was consistent with the previous multiple linear regression model. Furthermore, given the multicollinearity among Δ₁ₘ-WC, BMI₀, and Δ₁ₘ-BMI, two separate models were utilized to reduce potential bias. The mediation model for Δ₁ₘ-WC—adjusted for age, sex, SUA₀, eGFR₀, TyG₀, Δ₁ₘ-eGFR, Δ₁ₘ-TyG, TG₀, and HDL-C₀—yielded an R^2^ of 0.418, which was highly consistent with the R^2^ of the primary model (0.423). No evidence of significant multicollinearity was observed (variance inflation factor [VIF] < 10).

### Mediation effect of Δ₁ₘ-eGFR on the relationship between eGFR₀ and Δ₁ₘ-SUA

eGFR₀ significantly predicted Δ₁ₘ-eGFR (B = 0.22, *p* < 0.001), and Δ₁ₘ-eGFR significantly predicted Δ₁ₘ-SUA (B = −3.54, *p* < 0.001). The indirect effect was significant (B = −0.79, 95% CI: −1.33 to −0.28) (Fig. 3B, Table 2).

**Table 2:** Diagram of path coefficients for total, direct, and indirect effects

M		5,000 samplings				10,000 samplings			
		Effect	SE	LLCI	ULCI	Effect	SE	LLCI	ULCI
Δ_1m_-eGFR	Total effect	0.46	0.54	−0.60	1.53	0.46	0.54	−0.60	1.53
	Direct effect	1.24	0.52	0.21	2.27	1.24	0.52	0.21	2.27
	Indirect effect	−0.79	0.27	−1.33	−0.28	−0.78	0.27	−1.34	−0.29
Δ_1m_-BMI	Total effect	−1.03	1.19	−3.37	1.31	−1.03	1.19	−3.37	1.31
	Direct effect	2.59	1.41	−0.20	5.38	2.59	1.41	−0.20	5.38
	Indirect effect	−3.61	0.93	−5.61	−1.93	−3.62	0.93	−5.54	−1.91
Δ_1m_-WC (Without TWL_1_)	Total effect	−1.17	0.39	−1.93	−0.39	−1.17	0.39	−1.93	−0.39
	Direct effect	−0.29	0.44	−1.16	0.58	−0.29	0.44	−1.16	0.58
	Indirect effect	−0.87	0.26	−1.46	−0.40	−0.87	0.27	−1.45	−0.41
Δ_1m_-WC (With TWL_1_)	Total effect	−1.09	0.37	−1.82	−0.35	−1.09	0.37	−1.82	−0.35
	Direct effect	−0.86	0.54	−1.93	0.20	−0.86	0.54	−1.93	0.20
	Indirect effect	−0.22	0.41	−1.08	0.54	−0.22	0.42	−1.12	0.55
Δ_6m_-eGFR	Total effect	0.74	1.03	−1.36	2.85	0.74	1.03	−1.36	2.85
	Direct effect	−2.04	1.3	−4.70	0.62	−2.04	1.3	−4.70	0.62
	Indirect effect	2.78	1.26	0.37	5.37	2.78	1.26	0.39	5.31

### Mediation effect of Δ₁ₘ-BMI on the relationship between BMI₀ and Δ₁ₘ-SUA

BMI₀ significantly predicted Δ₁ₘ-BMI (B = 0.16, *p* < 0.001), and Δ₁ₘ-BMI significantly predicted Δ₁ₘ-SUA (B = −22.76, *p* < 0.001). The indirect effect was significant (B = −3.61, 95% CI: −5.61 to −1.93) (Fig. 3C, Table 2).

### Mediation effect of Δ₁ₘ-WC on the relationship between WC₀ and Δ₁ₘ-SUA

The mediation effect of Δ₁ₘ-WC was significantly moderated by TWL₁. In the absence of adjusting for TWL₁ (Fig. 3D, Table 2), WC₀ significantly and positively predicted Δ₁ₘ-WC (B = 0.09, *p* < 0.001), and Δ₁ₘ-WC significantly and negatively predicted Δ₁ₘ-SUA (B = −9.83, *p* < 0.001). The indirect effect was significant (B = −0.87, 95% CI: −1.46 to −0.40). Following adjustment for TWL₁, the indirect effect was no longer significant (Fig. 3E, Table 2).

### Stratified analyses of Δ₁ₘ-SUA Changes

1. Comparison Between EUA and NUA Groups

The NUA group had a significantly greater increase in Δ₁ₘ-SUA compared with the EUA group (44.48 vs. 1.90 μmol/L, *p* < 0.001; Fig. 3F).

2. Comparison by Δ₁ₘ-eGFR Levels.

Using the median Δ₁ₘ-eGFR (4.44 mL/min/1.73 m^2^) as the cutoff value, the group with Δ₁ₘ-eGFR < 4.44 had a mean decrease in Δ₁ₘ-SUA of 10.48 μmol/L, while the group with Δ₁ₘ-eGFR ≥ 4.44 had an average increase of 48.52 μmol/L (*p* < 0.001; Fig. 3G).

3. Comparison by Weight Loss Rate.

Using the median Δ₁ₘ-BMI (4.25 kg/m^2^) as the cutoff value, the slow weight loss group (Δ₁ₘ-BMI < 4.25 kg/m^2^) had a mean decrease in Δ₁ₘ-SUA of 6.76 μmol/L, whereas the rapid weight loss group (Δ₁ₘ-BMI ≥ 4.25 kg/m^2^) exhibited a mean increase of 44.25 μmol/L (*p* < 0.001; Fig. 3H).

### Factors influencing SUA₆ₘ After LSG

**Fig. 4:**
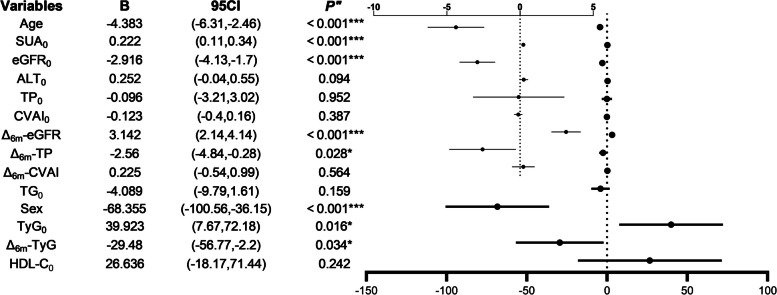
Forest plot of SUA₆ₘ independent predictors

**Fig. 5:**
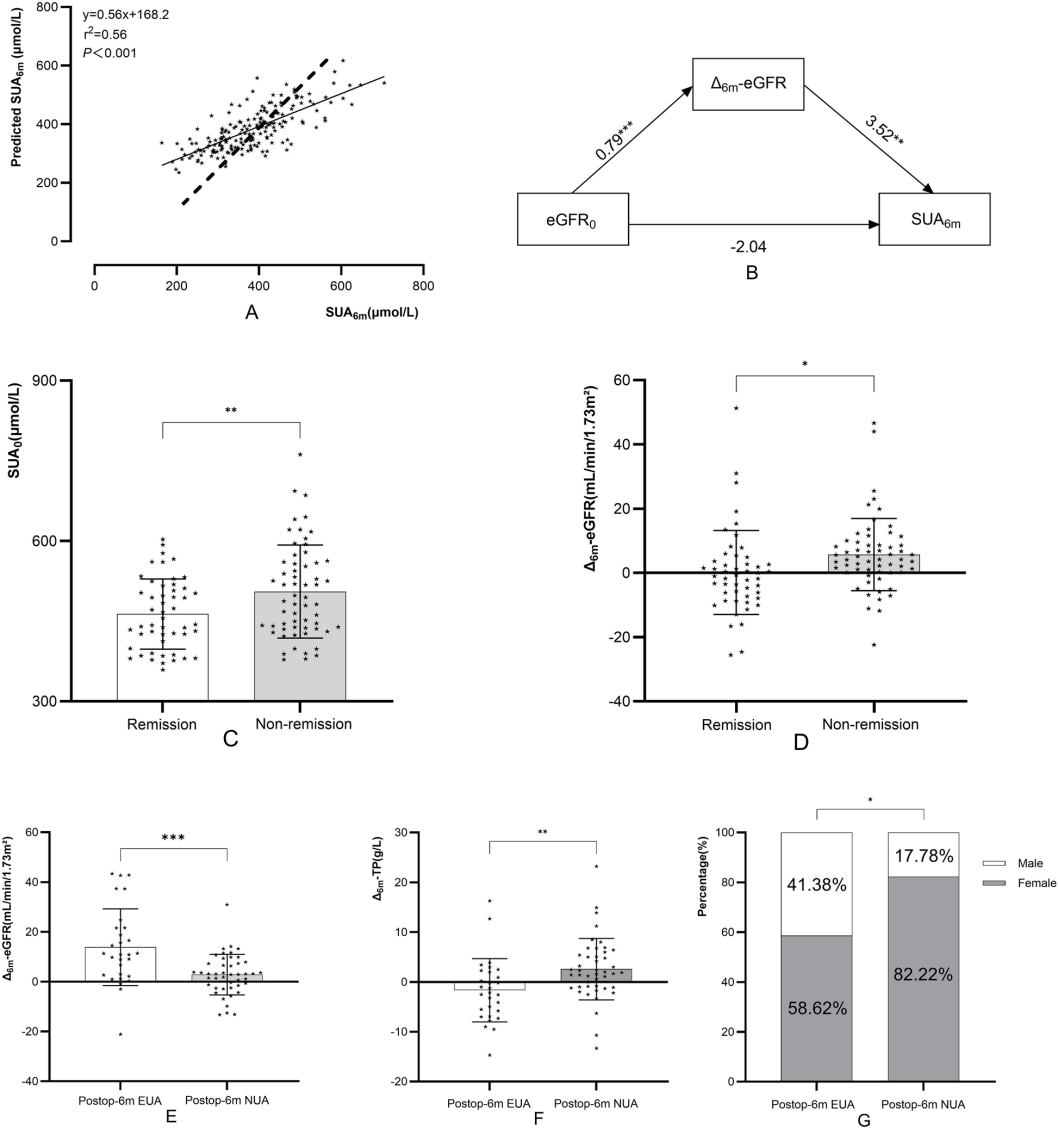
Multiple linear regression model fit for SUA_6m_ (**A**, The dashed line represents a slope of 1); Mediation effect of ∆_6m_-eGFR (**B**); Stratified comparisons in EUA group by SUA_6m_ remission (**C**-**D**); Stratified comparisons in NUA group by postop-6m SUA status **(E-G)**

Pearson or Spearman correlation analysis was utilized to identify factors correlated with SUA₆ₘ. Considering sample size constraints, variables with significant multicollinearity were excluded, and confounding factors (age, sex, TG₀, and HDL-C₀) were adjusted for. The final multiple linear regression model demonstrated (Fig. 4) that age, sex, SUA₀, TyG₀, eGFR₀, Δ₆ₘ-eGFR, Δ₆ₘ-TP, and Δ₆ₘ-TyG were independent predictors of SUA₆ₘ. The model explained 56% of the variance in SUA₆ₘ (R^2^ = 0.56; slope = 0.56, Fig. 5A).

### Mediation effect of Δ_6__ₘ_-eGFR on the relationship between eGFR₀ and SUA_6m_

eGFR₀ significantly predicted Δ_6ₘ_-eGFR (B = 0.79, *p* < 0.001), and Δ_6ₘ_-eGFR significantly predicted SUA_6ₘ_ (B = 3.52, *p* < 0.001). The indirect effect was significant (B = 2.78, 95% CI: 0.37 to 5.37) (Fig. 5B).

### Stratified analyses of SUA₆ₘ

#### EUA group stratification

The EUA group was stratified into a remission subgroup and a non-remission subgroup based on SUA₆ₘ normalization status. The remission subgroup had significantly lower SUA₀ levels compared with the non-remission subgroup (463.27 vs. 505.34 μmol/L, *p* < 0.01; Fig. 5C). Additionally, the remission subgroup had a smaller decrease in Δ₆ₘ-eGFR than the non-remission subgroup (0.14 vs. 5.69 mL/min/1.73 m^2^, *p* < 0.01; Fig. 5D).

#### NUA group stratification

The NUA group was stratified into a 6-month postoperative EUA (postop-6m EUA) subgroup (SUA₆ₘ exceeded the SUA threshold) and a 6-month postoperative NUA (postop-6m NUA) subgroup (SUA₆ₘ maintained normal). The postop-6m EUA subgroup had a larger decrease in Δ₆ₘ-eGFR compared with the postop-6m NUA subgroup (13.86 vs. 2.83 mL/min/1.73 m^2^, *p* < 0.01; Fig. 5E). Regarding Δ₆ₘ-total protein (TP), the postop-6m EUA subgroup showed an increase of 1.67 g/L, whereas the postop-6m NUA subgroup exhibited a decrease of 2.56 g/L (*p* < 0.01; Fig. 5F). Furthermore, the postop-6m EUA subgroup had a significantly higher proportion of males (60% vs. 31.84%, *p* < 0.05; Fig. 5G).

#### Postoperative SUA level changes by gender and baseline SUA status

**Fig. 6:**
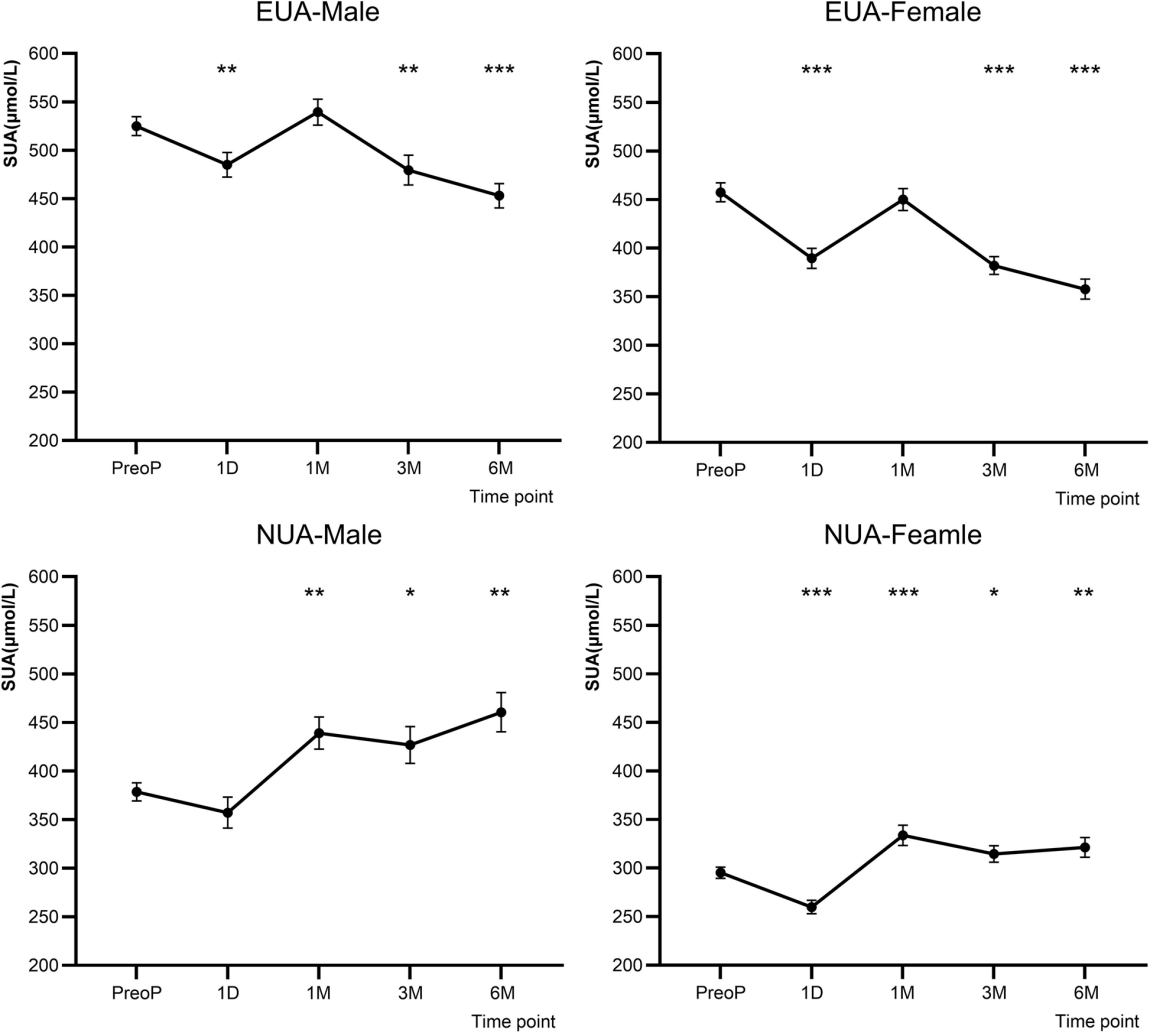
Postoperative SUA levels over time by gender and baseline SUA status

Patients were stratified into four subgroups based on gender and SUA₀: EUA males, EUA females, NUA males, and NUA females. In the overall group, SUA levels exhibited a significant decline beginning at 3 months postoperatively. In the NUA group, SUA₁ₘ, SUA₃ₘ, SUA₆ₘ were significantly higher than SUA₀. Among NUA group patients, males exhibited a significantly larger increase in Δ₆ₘ-SUA than females (81.86 vs. 26.19 μmol/L, *p* < 0.01) (Fig. 6).

## Discussion

Current research [14] has delineated the pathogenesis of gout into two key phases: first, deposition of monosodium urate (MSU) crystals under conditions of elevated SUA; second, activation of the inflammatory cascade that precipitates acute gout flares. While supraphysiologic SUA levels are requisite for MSU crystal nucleation, clinical observations demonstrate that only ~ 50% of patients with SUA levels ≥ 595 μmol/L develop gout over a 15-year period. This implies that disruption of endogenous biomolecular fiber architectures may serve as a pivotal prerequisite for MSU crystal deposition [15].

A study enrolling 70 patients with gout undergoing surgery under general anesthesia demonstrated a 44.3% short-term postoperative incidence of acute gout and a median onset time of 3.7 days [16]. Preoperative SUA ≥ 535.5 μmol/L and marked postoperative SUA fluctuations emerged as independent risk factors for acute flares. This process may parallel the "crystal dissolution reaction" observed during initial urate-lowering therapy (ULT): rapid SUA reduction can alter the physical or chemical properties of pre-existing MSU crystals, destabilizing microtubules and crystal aggregates on cartilage surfaces. Exposure of protein-depleted MSU crystals activates NLRP3 inflammasomes in monocytes and synovial cells, facilitating the secretion of proinflammatory cytokines, including interleukin-1β, and ultimately triggering acute gout flares [17]. Furthermore, a clinical study by Song et al. focusing on patients with gout following LSG further validated that continuous postoperative administration of ULT can effectively reduce the magnitude of postoperative SUA fluctuations, with a significantly lower incidence of acute gout flares compared to the non-treatment group [18]. Building on the aforementioned risk profiles, mechanisms, and clinical evidence, we propose the following optimized management strategy for the high-risk subgroup with rapid weight loss (Δ₁ₘ-BMI ≥ 4.25 kg/m^2^): 1. During the first postoperative month, SUA levels should be monitored weekly to dynamically capture the trajectory of urate fluctuations; 2. When SUA levels exceed 535.5 μmol/L (especially in patients with a history of gout), corresponding clinical interventions are indicated: concurrent administration of ULT and low-dose colchicine for prophylaxis. This strategy draws on the well-established approach for preventing acute flares during initial ULT in patients with chronic gout [17]. Through the dual effects of "stabilizing SUA levels + inhibiting inflammatory initiation," it precisely targets the core pathological processes underlying postoperative gout flares, ultimately achieving effective prevention and control of acute gout in high-risk patients following LSG.

Regarding the trend of SUA changes one month after MBS, existing findings remain conflicting: Wang et al. [19] drew specific conclusions via meta-analysis, whereas Yeo et al. reported contradictory results [20]. This discrepancy is likely closely linked to the high heterogeneity observed in both studies (I^2^ = 97% and 95%, respectively). Specifically, while both studies incorporated multiple metabolic and bariatric surgical approaches, they varied in procedural composition. Furthermore, Yeo’s study included a higher proportion of open surgeries, in contrast to Wang et al.’s study, which focused predominantly on laparoscopic procedures. Notably, Wang et al. mitigated heterogeneity through subgroup analysis (I^2^ reduced to 73%–81%), suggesting that surgical approach represents a major contributor to the observed high heterogeneity. Building on this, the present study focused exclusively on a single procedure (LSG), thereby offering targeted clinical insights to resolve the contradictory findings of the aforementioned meta-analyses.

While WC serves as a classic marker for evaluating visceral fat, theoretically, Δ_1m_-WC may modulate SUA levels via improvements in insulin resistance [21]—a property that could render it more clinically meaningful than weight metrics like Δ_1m_-BMI or TWL_1_. However, our findings demonstrate that after adjusting for TWL_1_, the independent effect of Δ_1m_-WC was effectively attenuated, with the previously significant mediating effect abating accordingly. Our results are consistent with those reported by Kankaya et al. [22]—namely, higher BMI₀ correlates with greater TWL₁. Additionally, the present study reveals that BMI₀ exerts an effect on Δ_1m_-SUA by mediating Δ_1m_-BMI. This underscores that in the early postoperative management of SUA, weight loss carries greater clinical relevance than isolated changes in waist circumference and should be prioritized during the development of clinical intervention strategies.

Per IFSO guidelines [23], patients who have undergone MBS should adhere to a high-protein, low-carbohydrate, and low-fat dietary pattern. With this dietary regimen, the body undergoes rapid lipolysis during the early postoperative period—a process further augmented by minimal carbohydrate intake. When acetyl-CoA generated from fat breakdown surpasses the metabolic capacity of the tricarboxylic acid cycle, ketone body production increases substantially. This, in turn, inhibits renal SUA excretion via multiple molecular mechanisms, ultimately resulting in elevated SUA levels.

The kidneys process UA via a complex system involving the coordinated action of multiple transporters: UA in the bloodstream is first filtered by the glomerulus into the tubular lumen. Approximately 90% of this filtered UA is taken up by the apical URAT1 transporter and subsequently reabsorbed into the circulation through the basolateral GLUT9a. Additionally, a subset of UA is secreted from the renal interstitial blood: this occurs via basolateral uptake by OAT1/OAT3, followed by apical efflux into the tubular lumen through ABCG2, NPT1, and NPT4. Under physiological conditions, reabsorptive and secretory processes are balanced, resulting in a net UA excretion equivalent to ~ 10% of the initial glomerular filtration load [24–26].

Elevated ketone body levels—organic anions—competitively bind to OAT1/OAT3 binding sites, directly inhibiting renal UA excretion. This mechanism was validated in the seminal 1965 study by Lecocq & McPhaul [27], which demonstrated that ketone body infusion reduces human uric acid clearance by 30%–50%. Concurrently, ketosis-induced acidosis alters the body’s pH environment. As a pH-sensitive UA efflux transporter, ABCG2 function is markedly impaired under acidic conditions [24, 26], further diminishing UA excretion. Additionally, UA transporter variants are not to be overlooked. The ABCG2 Q141K variant has a prevalence of 30% in East Asian populations, and the ABCG2 gene plays a critical role in intestinal UA excretion. When this gene is mutated, the intestine’s compensatory capacity for UA excretion is significantly impaired: homozygous individuals become highly reliant on renal function for UA excretion, and if renal excretion is compromised (e.g., during ketosis), their SUA levels rise more profoundly; heterozygous individuals, by contrast, retain partial ABCG2 transport function, resulting in relatively milder SUA fluctuations.

SLC2A9 variants alter UA transmembrane transport efficiency: when SLC2A9 variants enhancing reabsorption coexist with ABCG2 defects, the concomitant "reduced excretion and increased reabsorption" leads to marked SUA elevation. Conversely, SLC2A9 variants impairing function partially offset ABCG2 defects, yielding a blunted SUA response. URAT1 variants modulate renal reabsorption efficiency by altering protein affinity for UA, acting synergistically with ABCG2 and SLC2A9 variants to further amplify interindividual variability in SUA responses. Thus, the heterogeneous changes (elevation or reduction) in SUA levels after LSG in the present study may be partially attributed to the aforementioned genotypic differences [28, 29]. Regrettably, the absence of subject-specific genotypic data in this study precludes the ability to provide novel mechanistic insights, which constitutes one limitation of the current research.

The progression of obesity-related kidney injury can be categorized into three stages [30]: the ultrafiltration phase, normal filtration phase, and hypofiltration phase. After MBS, patients with obesity exhibit stabilized or improved eGFR, along with marked reductions in urinary protein levels—even pathological renal damage may be attenuated. A meta-analysis [31] demonstrated that among patients with baseline eGFR < 90 mL/min/1.73 m^2^, postoperative eGFR increased by 13.81 mL/min/1.73 m^2^ (95% CI: 10.31–17.32), whereas in those with baseline eGFR > 120 mL/min/1.73 m^2^, eGFR decreased by 9.61 mL/min/1.73 m^2^ (95% CI: −16.31 to −2.91). Notably, MBS approaches did not exert a significant impact on changes in eGFR. Similarly, Moriconi et al. [32] observed postoperative eGFR decline in patients with preoperative renal hyperfiltration, while those with baseline eGFR < 90 mL/min/1.73 m^2^ showed improvements—findings consistent with the present study. Crucially, postoperative eGFR reduction does not signify renal dysfunction but rather reflects resolution of renal hyperfiltration, indicating ongoing renal function recovery. Therefore, optimizing preoperative eGFR and monitoring/intervening in postoperative eGFR dynamics represent key strategies for modulating postoperative SUA levels. Furthermore, mediation analysis identified the mechanistic pathway "eGFR₀ → Postoperative eGFR change → SUA," suggesting clinicians may indirectly regulate SUA by preserving postoperative renal function—for instance, through adequate postoperative fluid resuscitation and avoidance of nephrotoxic medications—to minimize the magnitude of postoperative eGFR decline.

These findings provide a framework for stratified management: For patients with baseline renal impairment (eGFR: 60 ~ 89 mL/min/1.73m^2^), prioritization of etiological interventions (e.g., diabetes control, improvement of renal perfusion) is essential to optimize renal function preoperatively, thereby preventing postoperative deterioration that could adversely affect SUA; For patients with baseline renal hyperfiltration(eGFR > 125 mL/min/1.73m^2^), postoperative eGFR decline reduces renal SUA clearance. Clinicians should closely monitor dynamic changes in eGFR and SUA in this subgroup and initiate targeted interventions in a timely manner.

A major limitation of the present study is the lack of systematic collection of detailed dietary information. All patients were advised to consume 60–80 g of whey protein daily for 6 months postoperatively, as this low-purine, high-quality protein exerts minimal potential impact on SUA levels. However, due to inadequate documentation of patients’ overall dietary patterns—including the potential intake of high-purine animal proteins commonly observed in postoperative metabolic and bariatric diets—we cannot rule out the potential impact of dietary purine load (independent of the study-recommended whey protein) on the 44.25 μmol/L elevation in Δ1m-SUA observed in the rapid weight loss group. Additionally, it remains unclear whether the divergent trends—Δ_6m_-TP elevation in the Postop-6m EUA group versus a decreasing trend in the Postop-6m NUA group—are associated with postoperative dietary patterns.

Sex hormones play a critical role in regulating SUA metabolic homeostasis: Estrogen reduces UA reabsorption by downregulating SLC2A9 expression and modulates the cellular localization and stability of ABCG2 via estrogen receptor signaling pathways, thereby promoting UA excretion—a key mechanism underlying the lower baseline SUA levels in women [33]. Post-LSG reduction in adipose tissue induces alterations in sex hormone levels: increased postoperative testosterone secretion in males selectively upregulates Smct1, enhancing the driving force for renal UA reabsorption [34, 35]. This may represent a potential mechanism explaining the more pronounced postoperative SUA elevation in the male NUA group. However, sex hormone-related assays were not included in the study’s assessment panel, precluding the integration of sex hormone levels and their regulatory effects on UA metabolism into statistical analyses. This constitutes another major limitation of the present research.

Additionally, the single-center retrospective observational design, focus on a single surgical modality, modest sample size of 184 patients, and inherent heterogeneity between preoperative groups may restrict the generalizability of the findings. Furthermore, as a retrospective exploratory analysis without statistical correction for multiple comparisons, this study confers an elevated risk of Type I errors.

Future research directions should include:1. Prospective studies to systematically record postoperative dietary purine content, thereby excluding the confounding influence of diet on postoperative SUA changes; 2. Incorporation of genetic analyses (ABCG2, SLC2A9, and URAT1 genotyping) to explore the impact of distinct genotypes on SUA trajectories after LSG; 3. Investigation of associations between post-LSG SUA changes and alterations in relevant hormonal profiles (estrogen, testosterone, leptin, adiponectin) as well as ketone body levels, which represents a promising future research avenue; 4. Initiation of prospective randomized controlled trials (RCTs) of preoperative prophylactic ULT in patients with rapid postoperative weight loss (e.g., Δ_1m_-BMI ≥ 4.25 kg/m^2^) to determine whether prophylactic urate reduction can attenuate the rate of postoperative SUA fluctuation and mitigate the risk of acute gout flares.

## Conclusion

This study draws the following key conclusions: (1) Δ₁ₘ-BMI is a critical factor influencing Δ₁ₘ-SUA. For patients with rapid weight loss (Δ₁ₘ-BMI ≥ 4.25 kg/m^2^), weekly SUA monitoring within the first postoperative month is recommended to promptly identify uric acid fluctuations. Prompt clinical intervention is necessary when SUA exceeds 535.5 μmol/L, especially in patients with a history of gout. (2) Preoperative optimization of renal reserve is advised for patients with impaired baseline renal function. For those with baseline renal hyperfiltration, clinical monitoring should prioritize postoperative eGFR and SUA changes, with timely targeted interventions if indicated. (3) Male patients in the NUA group are at higher risk of SUA elevation at 6 months postoperatively; clinicians should tailor dietary and lifestyle modifications to dynamic monitoring findings, and consider pharmacologic intervention if necessary to maintain uric acid homeostasis.

Additionally, this study identifies age and SUA₀ as independent predictors of Δ₁ₘ-SUA, while age, sex, SUA₀, TyG₀, Δ₆ₘ-TP, and Δ₆ₘ-TyG are independent predictors of SUA₆ₘ.

## Supplementary Information

Supplementary Material 1.

## Data Availability

The datasets used and analysed during the current study are available from the corresponding author on reasonable request.

## Abbreviations

NUA: Normal SUA
EUA: Elevated SUA
BMI: Body mass index
WC: Waist circumference
SUA: Serum uric acid
Cre: Serum creatinine
eGFR: Estimated glomerular filtration rate
TP: Total protein
TyG: Triglyceride-glucose index
FBG: Fasting blood glucose
TG: Triglycerides
TC: Total cholesterol
HDL-C: High-density lipoprotein cholesterol
LDL-C: Low-density lipoprotein cholesterol
CVAI: Chinese Visceral Adiposity Index
ALT: Alanine aminotransferase
AST: Aspartate aminotransferase
EWL: Excess weight loss
TWL: Total weight loss
POD: Postoperative day
HUA: Hyperuricemia
MBS: Metabolic and bariatric surgery
LSG: Laparoscopic Sleeve Gastrectomy

## References

[1] World Obesity Federation. World Obesity Atlas 2025. London: World Obesity Federation; 2025.

[2] Kivimäki M, Strandberg T, Pentti J, et al. Body-mass index and risk of obesity-related complex multimorbidity: an observational multicohort study. Lancet Diabetes Endocrinol. 2022;10(4):253–63. 10.1016/s2213-8587(22)00033-x. 35248171 10.1016/S2213-8587(22)00033-X PMC8938400

[3] Zeng J, Lawrence WR, Yang J, et al. Association between serum uric acid and obesity in Chinese adults: a 9-year longitudinal data analysis. BMJ Open. 2021;11(2):e041919. 10.1136/bmjopen-2020-041919. 33550245 10.1136/bmjopen-2020-041919 PMC7908911

[4] Cheang C, Law S, Ren J, Chan W, Wang C, Dong Z. Prevalence of hyperuricemia in patients with severe obesity and the relationship between serum uric acid and severe obesity: a decade retrospective cross-section study in Chinese adults. Front Public Health. 2022;10:986954. 10.3389/fpubh.2022.986954. 36091568 10.3389/fpubh.2022.986954 PMC9462510

[5] Du L, Zong Y, Li H, et al. Hyperuricemia and its related diseases: mechanisms and advances in therapy. Signal Transduct Target Ther. 2024;9(1):212. 10.1038/s41392-024-01916-y. 39191722 10.1038/s41392-024-01916-y PMC11350024

[6] Chinese Multidisciplinary Expert Consensus on the Diagnosis and Treatment of Hyperuricemia and Related Diseases. Chin Med J. 2017;130(20):2473-2488. 10.4103/0366-6999.216416 10.4103/0366-6999.216416 PMC5684625 29052570

[7] Mills DW, Woolley DM, Ammori BJ, Chinoy H, Syed AA. Changes in serum urate levels after bariatric surgery in patients with obesity: an observational study. Obes Surg. 2024;34(5):1737–41. 10.1007/s11695-024-07191-8. 38528214 10.1007/s11695-024-07191-8 PMC11031430

[8] Chu Y, Cao C, Shao Y, Hua R, Yao Q. Excess weight loss at 6 months following laparoscopic sleeve gastrectomy correlates with the remission of hyperuricemia. Obes Surg. 2025;35(3):829–36. 10.1007/s11695-025-07668-0. 39810032 10.1007/s11695-025-07668-0

[9] Xu C, Wen J, Yang H, et al. Factors influencing early serum uric acid fluctuation after bariatric surgery in patients with hyperuricemia. Obes Surg. 2021;31(10):4356–62. 10.1007/s11695-021-05579-4. 34309788 10.1007/s11695-021-05579-4

[10] Katsogridaki G, Tzovaras G, Sioka E, et al. Hyperuricemia and acute gout after laparoscopic sleeve gastrectomy. Clin Obes. 2019;9(2):e12296. 10.1111/cob.12296. 30815983 10.1111/cob.12296

[11] Levey AS, Stevens LA, Schmid CH, et al. A new equation to estimate glomerular filtration rate. Ann Intern Med. 2009;150(9):604–12. 10.7326/0003-4819-150-9-200905050-00006. 19414839 10.7326/0003-4819-150-9-200905050-00006 PMC2763564

[12] Lewis TV, Harrison DL, Gildon BL, Carter SM, Turman MA. Applicability of the Schwartz equation and the chronic kidney disease in children bedside equation for estimating glomerular filtration rate in overweight children. Pharmacotherapy. 2016;36(6):598–606. 10.1002/phar.1763. 27138894 10.1002/phar.1763

[13] Fritz MS, Mackinnon DP. Required sample size to detect the mediated effect. Psychol Sci. 2007;18(3):233–9. 10.1111/j.1467-9280.2007.01882.x. 17444920 10.1111/j.1467-9280.2007.01882.x PMC2843527

[14] Shi C, Zhou Z, Chi X, et al. Recent advances in gout drugs. Eur J Med Chemistry. 2023;245(Pt 1):114890. 10.1016/j.ejmech.2022.114890. 10.1016/j.ejmech.2022.114890 36335742

[15] Zhang WZ. Why does hyperuricemia not necessarily induce gout? Biomolecules. 2021;11(2). 10.3390/biom11020280. 33672821 10.3390/biom11020280 PMC7918342

[16] Jeong H, Jeon CH. Clinical characteristics and risk factors for gout flare during the postsurgical period. Adv Rheumatol. 2019;59(1):31. 10.1186/s42358-019-0075-7. 31345250 10.1186/s42358-019-0075-7

[17] Schlesinger N. Treatment of chronic gouty arthritis: it is not just about urate-lowering therapy. Semin Arthritis Rheum. 2012;42(2):155–65. 10.1016/j.semarthrit.2012.03.010. 22542277 10.1016/j.semarthrit.2012.03.010

[18] Song K, He M, Kong X, et al. Benefits of uric acid-lowering medication after bariatric surgery in patients with gout. BMC Surg. 2024;24(1):186. 10.1186/s12893-024-02472-6. 38877436 10.1186/s12893-024-02472-6 PMC11177500

[19] Wang G, Hui Y, Wang F, et al. Meta-analysis of the effect of bariatric metabolic surgery on serum uric acid levels in obese patients. Chin J Obes Metabol Dis. 2024;10(1):45–57. 10.3877/cma.j.issn.2095-9605.2024.01.008.

[20] Yeo C, Kaushal S, Lim B, et al. Impact of bariatric surgery on serum uric acid levels and the incidence of gout-a meta-analysis. Obes Rev. 2019;20(12):1759–70. 10.1111/obr.12940. 31468681 10.1111/obr.12940

[21] Klein S, Gastaldelli A, Yki-Järvinen H, Scherer PE. Why does obesity cause diabetes? Cell Metab. 2022;34(1):11–20. 10.1016/j.cmet.2021.12.012. 34986330 10.1016/j.cmet.2021.12.012 PMC8740746

[22] Kankaya B, Buyukasik S, Altundal YE, et al. Weight loss dynamics after laparoscopic sleeve gastrectomy: a retrospective single center analysis with age and preoperative weight stratification. Sci Rep. 2025;15(1):8771. 10.1038/s41598-025-93826-4. 40082531 10.1038/s41598-025-93826-4 PMC11906818

[23] Eisenberg D, Shikora SA, Aarts E, et al. 2022 American Society for Metabolic and Bariatric Surgery (ASMBS) and International Federation for the Surgery of Obesity and Metabolic Disorders (IFSO): Indications for Metabolic and Bariatric Surgery. Surg Obes Related Dis. 2022;18(12):1345–56. 10.1016/j.soard.2022.08.013. 10.1016/j.soard.2022.08.013 36280539

[24] Nigam SK, Bhatnagar V. The systems biology of uric acid transporters: the role of remote sensing and signaling. Curr Opin Nephrol Hypertens. 2018;27(4):305–13. 10.1097/mnh.0000000000000427. 29847376 10.1097/MNH.0000000000000427 PMC6275126

[25] Sun HL, Wu YW, Bian HG, et al. Function of uric acid transporters and their inhibitors in hyperuricaemia. Front Pharmacol. 2021;12:667753. 10.3389/fphar.2021.667753. 34335246 10.3389/fphar.2021.667753 PMC8317579

[26] Ristic B, Sikder MOF, Bhutia YD, Ganapathy V. Pharmacologic inducers of the uric acid exporter ABCG2 as potential drugs for treatment of gouty arthritis. Asian J Pharm Sci. 2020;15(2):173–80. 10.1016/j.ajps.2019.10.002. 32373197 10.1016/j.ajps.2019.10.002 PMC7193448

[27] Lecocq FR, McPhaul JJ Jr. The effects of starvation, high fat diets, and ketone infusions on uric acid balance. Metabolism. 1965;14:186–97. 10.1016/s0026-0495(65)80039-7. 14261402 10.1016/s0026-0495(65)80039-7

[28] Woodward OM, Köttgen A, Coresh J, Boerwinkle E, Guggino WB, Köttgen M. Identification of a urate transporter, ABCG2, with a common functional polymorphism causing gout. Proc Natl Acad Sci U S A. 2009;106(25):10338–42. 10.1073/pnas.0901249106. 19506252 10.1073/pnas.0901249106 PMC2700910

[29] Ichida K, Matsuo H, Takada T, et al. Decreased extra-renal urate excretion is a common cause of hyperuricemia. Nat Commun. 2012;3:764. 10.1038/ncomms1756. 22473008 10.1038/ncomms1756 PMC3337984

[30] Kambham N, Markowitz GS, Valeri AM, Lin J, D’Agati VD. Obesity-related glomerulopathy: an emerging epidemic. Kidney Int. 2001;59(4):1498–509. 10.1046/j.1523-1755.2001.0590041498.x. 11260414 10.1046/j.1523-1755.2001.0590041498.x

[31] Huang H, Lu J, Dai X, et al. Improvement of renal function after bariatric surgery: a systematic review and meta-analysis. Obes Surg. 2021;31(10):4470–84. 10.1007/s11695-021-05630-4. 34355340 10.1007/s11695-021-05630-4

[32] Moriconi D, Nannipieri M, Dadson P, Rosada J, Tentolouris N, Rebelos E. The beneficial effects of bariatric-surgery-induced weight loss on renal function. Metabolites. 2022;12(10). 10.3390/metabo12100967. 36295869 10.3390/metabo12100967 PMC9608617

[33] Halperin Kuhns VL, Woodward OM. Sex differences in urate handling. Int J Mol Sci. 2020;21(12). 10.3390/ijms21124269. 32560040 10.3390/ijms21124269 PMC7349092

[34] Sarwer DB, Spitzer JC, Wadden TA, et al. Sexual functioning and sex hormones in persons with extreme obesity and seeking surgical and nonsurgical weight loss. Surg Obes Relat Dis. 2013;9(6):997–1007. 10.1016/j.soard.2013.07.003. 24120985 10.1016/j.soard.2013.07.003 PMC3864660

[35] Hosoyamada M, Takiue Y, Shibasaki T, Saito H. The effect of testosterone upon the urate reabsorptive transport system in mouse kidney. Nucleosides Nucleotides Nucleic Acids. 2010;29(7):574–9. 10.1080/15257770.2010.494651. 20589576 10.1080/15257770.2010.494651

