# Supplementary material for: Short-term dynamics of serum uric acid and its influencing factors in patients with obesity after laparoscopic sleeve gastrectomy
Source: BMC Surg. 2025 Dec 23;26:75. doi: 10.1186/s12893-025-03437-z (PMC12836822; doi:10.1186/s12893-025-03437-z)
Supplement: Supplementary file 1 — Supplementary Material 1. [file 12893_2025_3437_MOESM1_ESM.pdf]

STROBE Checklist

Title and abstract

(P1) 1a Indicate the study's design with a commonly used term in the title or the abstract

Methods: We performed a retrospective analysis of data from 184 patients with obesity who underwent laparoscopic sleeve gastrectomy (LSG).

(P1~2) 1b Provide in the abstract an informative and balanced summary of what was done and what was found

Background: The pathophysiological association between obesity and hyperuricemia (HUA) is well-established. Metabolic and bariatric surgery (MBS) has been shown to effectively manage severe obesity and lead to sustained reductions in serum uric acid (SUA) over the long term; however, the factors modulating short-term fluctuations in SUA (i.e., within 6 months postoperatively) and their underlying mechanisms remain poorly elucidated.

Methods: We performed a retrospective analysis of data from 184 patients with obesity who underwent laparoscopic sleeve gastrectomy (LSG). Clinical data were retrieved at baseline, on postoperative day 1, and at 1, 3, and 6 months postoperatively. Per established guidelines, patients were stratified into a normal SUA (NUA) group (n=74) and an elevated SUA (EUA) group (n=110).

Results: Age, baseline estimated glomerular filtration rate ($eGFR_0$), baseline SUA (SUA_0), 1-month postoperative change in eGFR (Δ_{1m}-eGFR), and 1-month postoperative change in BMI (Δ_{1m}-BMI) were independent predictors of 1-month postoperative change in SUA (Δ_{1m}-SUA).

Age, sex, SUA_0, baseline triglyceride-glucose index (TyG_0), $eGFR_0$, 6-month

postoperative change in eGFR (Δ_{6m}-eGFR), 6-month postoperative change in total protein (Δ_{6m}-TP), and 6-month postoperative change in TyG (Δ_{6m}-TyG) were independent predictors of 6-month postoperative SUA (SUA_{6m}).

Process-mediated mediation analysis revealed that the effect of baseline BMI (BMI_0) on Δ_{1m}-SUA was fully mediated by Δ_{1m}-BMI; the effect of $eGFR_0$ on Δ_{1m}-SUA was partially mediated by Δ_{1m}-eGFR, while the effect of $eGFR_0$ on SUA_{6m} was fully mediated by Δ_{6m}-eGFR.

In the EUA group, 3-month postoperative SUA (SUA_{3m}) was significantly lower than the baseline value. In the NUA group, SUA at 1, 3, and 6 months postoperatively (SUA_{1m}/SUA_{3m}/SUA_{6m}) remained significantly higher than the baseline value, with a more pronounced increase in Δ_{6m}-eGFR noted in males compared to females.

Conclusion: Δ_{1m}-BMI is a key determinant of Δ_{1m}-SUA. Patients with Δ_{1m}-BMI $\geq$ 4.25 kg/m^2 warrant weekly SUA monitoring during the first month postoperatively. Prompt clinical intervention is necessary when SUA exceeds 535.5 μmol/L, especially in patients with a history of gout. For patients with baseline impaired renal function (eGFR: 60–89 mL/min/1.73m^2), preoperative optimization of renal reserve is recommended; those with renal hyperfiltration (eGFR > 125 mL/min/1.73m^2) require intensified postoperative monitoring of eGFR and SUA. Males in the NUA group are at an increased risk of SUA elevation at 6 months postoperatively. Furthermore, age and SUA_0 are independent predictors of Δ_{1m}-SUA, while age, sex, SUA_0, TyG_0, Δ_{6m}-TP, and Δ_{6m}-TyG are independent predictors of SUA_{6m}.

Key words: Hyperuricemia, Laparoscopic Sleeve Gastrectomy

Introduction

(P2~3) 2 Explain the scientific background and rationale for the investigation being

reported

According to projections from the World Obesity Atlas 2025, over 1.1 billion people worldwide will be living with obesity by 2030. Obesity is not only a major risk factor for type 2 diabetes, cardiovascular disease, and malignant tumors but also closely linked to the development of HUA. Furthermore, persistent HUA further elevates the risk of gout, cardiovascular events, and chronic kidney disease.

Current first-line medications for HUA and gout in China are associated with multiple adverse effects, which limits their widespread clinical use. MBS not only achieves long-term stable weight loss but also significantly improves SUA metabolism. However, existing studies indicate that in the early postoperative period (especially within the first month), patients experience marked fluctuations in SUA levels—accompanied by a parallel increase in the risk of acute gout flares. Currently, there is a paucity of systematic studies on the factors modulating short-term (particularly within 1 month) SUA fluctuations in patients with obesity undergoing LSG. Furthermore, the first six months after MBS constitute a critical period of rapid weight loss and serve as a key milestone for efficacy evaluation; yet the patterns of SUA metabolic alterations during this period remain incompletely characterized.

(P3) 3 State specific objectives, including any prespecified hypotheses

Given this research gap, this retrospective analysis was designed to address two core objectives: First, it focuses on the early postoperative period (within 1 month) to identify factors modulating short-term SUA fluctuations, with the goal of guiding targeted perioperative monitoring. Second, it examines the critical 6-month postoperative time point, systematically analyzing factors associated with SUA levels at this stage to offer clinical insights that inform

the optimization of long-term metabolic management strategies.

Methods

(P3) 4 Present key elements of study design early in the paper

5 Describe the setting, locations, and relevant dates, including periods of recruitment, exposure, follow-up, and data collection

This retrospective observational study of patients with obesity who underwent MBS at our institution (2022–2024) involved no therapeutic interventions, with data solely retrieved from our institutional medical record system in July 2025.

(P4) 6a Give the eligibility criteria, and the sources and methods of selection of participants. Describe methods of follow-up

Inclusion criteria were: (1) Patients undergoing their first MBS via the LSG approach; (2) age between 16 and 65 years; (3) complete clinical data were available. Exclusion criteria were: (1) use of urate-lowering medications within 1 month prior to surgery or during follow-up; (2) development of major postoperative complications. Based on these criteria, we retrieved clinical data from 184 patients with obesity who underwent LSG at our hospital between 2022 and 2024 (Figure 1). SUA levels were defined per the Chinese Guidelines for Diagnosis and Treatment of Hyperuricemia and Gout (2023), with thresholds set at >420 μmol/L for males and >357 μmol/L for females. Patients were stratified into the NUA group (n=74) and the EUA group (n=110).

(p4) 7 Clearly define all outcomes, exposures, predictors, potential confounders, and effect modifiers. Give diagnostic criteria, if applicable Data sources/measurement

Outcome: SUA levels; exposure: LSG.

Diagnostic criteria: SUA levels were defined per the Chinese Guidelines for Diagnosis and Treatment of Hyperuricemia and Gout (2023), with thresholds set at >420 μmol/L for males and >357 μmol/L for females.

(P4~5) 8* For each variable of interest, give sources of data and details of methods of assessment (measurement). Describe comparability of assessment methods if there is more than one group

Case data were retrospectively retrieved at baseline, on postoperative day 1 (POD 1), and at 1, 3, and 6 months postoperatively. Retrieved indicators included height (cm), weight (kg), body mass index (BMI, kg/m^2), waist circumference (WC, cm), SUA (μmol/L), serum creatinine (SCr, μmol/L), total protein (TP, g/L), triglyceride-glucose index (TyG), fasting blood glucose (FBG, mmol/L), triglycerides (TG, mmol/L), total cholesterol (TC, mmol/L), high-density lipoprotein cholesterol (HDL-C, mmol/L), low-density lipoprotein cholesterol (LDL-C, mmol/L), Chinese visceral adiposity index (CVAI), alanine aminotransferase (ALT, U/L), aspartate aminotransferase (AST, U/L), and apolipoprotein A1 (Apo A1, g/L). Given the limited accuracy of measurements during early postoperative recovery, patient weight, BMI, WC were not recorded on POD 1.

Key derived variables were calculated using the following formulas: For patients aged >18 years: The CKD-EPI equation[11] was applied: $eGFR=141\times\min(Scr(mg/dL)/\kappa,1)^{-\alpha}\times\max(Scr(mg/dL)/\kappa,1)^{-1.209}\times0.993^{Age}\times1.018[\text{if female}]\times1.159[\text{if black}]$ (κ= 0.7 for females, 0.9 for males; α= -0.329 for females, -0.411 for males); For adolescent patients aged 16-17 years: The Schwartz equation[12] was used: $eGFR = 0.55 \times Height\ (cm) / SCr\ (mg/dL)$

Excess Weight Loss Percentage (EWL%): [(Baseline BMI – Follow-up BMI) / (Baseline

BMI – 25)] × 100% (with an ideal BMI set at 25 kg/m²)

Total Weight Loss Percentage (TWL%): [(Baseline Weight – Follow-up Weight) / Baseline Weight] × 100%

Triglyceride-Glucose Index (TyG): ln [TG (mg/dL) × FBG (mg/dL) / 2]

Chinese Visceral Adiposity Index (CVAI):Male:-267.93+0.68×Age+0.03×BMI (kg/m²)+4.00×WC(cm)+22.00×lg （TG, mmol/L） -16.32×HDL-C(mmol/L); Female: 187.32+1.71×Age+4.32×BMI+1.12×WC+39.76×lg（TG）-11.66×HDL-C

(P7-8) 9 Describe any efforts to address potential sources of bias

Baseline characteristics of the EUA and NUA groups are summarized in Table 1. Statistically significant differences between the two groups were observed in age, sex, SCr, TG, HDL-C, and Apo A1. Given the limited sample size of each group when analyzed independently—which may reduce statistical power—the two cohorts were combined for comprehensive statistical analyses to evaluate the impact of LSG on SUA levels in the overall study population.

10 Explain how the study size was arrived at

(P4) Based on these criteria, we retrieved clinical data from 184 patients with obesity who underwent LSG at our hospital between 2022 and 2024.

(P9, 13) In the multiple regression analysis, 12–14 variables were included, and the sample size (n=184) was deemed appropriate.

(P10) To validate the aforementioned mediating pathways, a simple mediation model was constructed using the PROCESS macro (developed by Hayes). Following standard guidelines proposed by Fritz & MacKinnon (2007)[13], we preset moderate effect sizes (path α=0.39 for

X→M and path β=0.39 for M→Y), a significance level of 0.05, and a target power of 0.80. This yields a minimum effective sample size of 71 cases required to detect mediation effects. With a total sample size of 184 in the current study, this requirement was satisfied.

11 Explain how quantitative variables were handled in the analyses. If applicable, describe which groupings were chosen and why

(P4) Patients were stratified into the NUA group (n=74) and the EUA group (n=110).

(P11) Using the median Δ_{1m}-eGFR (4.44 mL/min/1.73 m^2) as the cutoff value, the group with Δ_{1m}-eGFR < 4.44 had a mean decrease in Δ_{1m}-SUA of 10.48 μmol/L, while the group with Δ_{1m}-eGFR ≥ 4.44 had an average increase of 48.52 μmol/L ($p < 0.001$; Figure 3G).

(P12) Using the median Δ_{1m}-BMI (4.25 kg/m^2) as the cutoff value, the slow weight loss group (Δ_{1m}-BMI < 4.25 kg/m^2) had a mean decrease in Δ_{1m}-SUA of 6.76 μmol/L, whereas the rapid weight loss group (Δ_{1m}-BMI ≥ 4.25 kg/m^2) exhibited a mean increase of 44.25 μmol/L ($p < 0.001$; Figure 3H).

12a Describe all statistical methods, including those used to control for confounding

(P6~7) Statistical analyses were performed using SPSS version 27.0. Categorical variables are presented as frequencies (n, %). The normality of continuous variables was evaluated via the Kolmogorov-Smirnov test: normally distributed continuous variables are expressed as mean ± standard deviation (SD), while non-normally distributed variables are reported as median (interquartile range). Chi-square tests were utilized for comparing categorical variables. For normally distributed continuous variables, independent samples t-tests were conducted, with Pearson's correlation coefficient (r) used to assess associations; for non-normally distributed continuous variables, Mann-Whitney U tests were employed, with

Spearman's correlation coefficient (ρ) for evaluating associations. Mediation analyses were performed using the PROCESS macro for SPSS. Multiple linear regression analysis was used to identify independent influencing factors. Repeated measures analysis of variance was applied to compare preoperative and postoperative SUA levels. A two-tailed p-value < 0.05 was considered statistically significant.

13* (a) Report numbers of individuals at each stage of study—eg numbers potentially eligible, examined for eligibility, confirmed eligible, included in the study, completing follow-up, and analysed (b) Give reasons for non-participation at each stage (c) Consider use of a flow diagram

(P7) Initially, a total of 1988 patients with obesity who underwent MBS at our institution between 2022 and 2024 were identified as the starting cohort. A stepwise exclusion process was performed to refine the study population, as outlined below:

First, 2 patients were excluded because their ages were outside the 16–65-year range, leaving a final sample size of 1986. Subsequently, 36 patients were excluded for undergoing non- LSG MBS or revision surgery, leaving 1950 individuals eligible for further assessment. Additionally, 85 patients were excluded because they used urate-lowering medications either 1 month preoperatively or during the follow-up period, reducing the cohort to 1865. Finally, 1681 patients were excluded due to incomplete baseline data or postoperative follow-up data.

After application of all exclusion criteria, 184 patients were ultimately included in the retrospective analysis as the final study cohort. The detailed patient screening and enrollment process is illustrated in Figure 1.

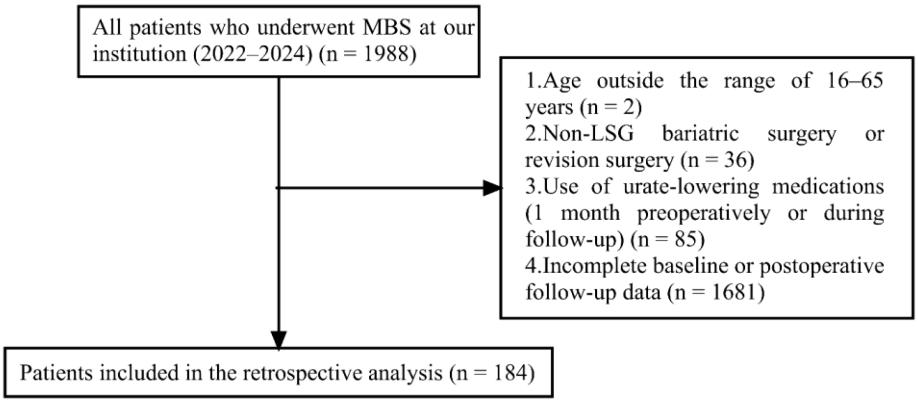

14* (a) Give characteristics of study participants (eg demographic, clinical, social) and information on exposures and potential confounders

(P8) Baseline characteristics of the EUA and NUA groups are summarized in Table 1. Statistically significant differences between the two groups were observed in age, sex, SCr, TG, HDL-C, and Apo A1. Given the limited sample size of each group when analyzed independently—which may reduce statistical power—the two cohorts were combined for comprehensive statistical analyses to evaluate the impact of LSG on SUA levels in the overall study population.

Variables	EUA	NUA	*χ2/t/Z*	*P''*
Age	32.28±8.95	36.31±7.48	-3.31	0.001**
Sex (Female%)	63(57.27)	54(72.97)	4.71	0.030*
Weight (kg)	118.12±24.59	110.9±26.41	1.9	0.059
BMI (kg/m²)	41.63±6.87	40.07±8.18	1.4	0.163
WC (cm)	124.33±13.69	121.24±15.25	1.43	0.154
SUA(μmol/L)	486.22±80.39	317.71±55.84	16.78	< 0.001***
SCr(μmol/L)	61.7±14.93	54.45±11.94	3.41	0.001**
eGFR(mL/min/1.73m²)	118.63±13.1	119.27±2.7	-0.34	0.736
TP(g/L)	74.33±4.62	73.04±4.62	1.86	0.064
TyG	9.14±0.76	9.07±0.86	0.55	0.583

FBG (mmol/L)	5.51(4.63, 6.86)	5.48(4.8, 7.59)	-0.71	0.479
TG (mmol/L)	1.8(1.37, 2.64)	1.48(1.16, 2.51)	-2.21	0.027*
TC (mmol/L)	5.35±1.17	5.41±1.06	-0.37	0.71
HDL-C(mmol/L)	1.17±0.23	1.31±0.29	-3.5	0.001**
LDL-C(mmol/L)	3.32±0.79	3.32±0.83	0.057	0.954
CVAI	212.47±64.11	195.36±66.76	1.75	0.082
Apo A_1(g/L)	1.39±0.26	1.48±0.24	-2.43	0.016*
ALT(U/L)	38.3(23.65, 59.25)	36.35(18.7,64.1)	-0.77	0.443

(P9) Considering sample size constraints, variables with significant multicollinearity were excluded, with adjustment for confounding factors including age, sex, baseline triglycerides (TG_0), and baseline high-density lipoprotein cholesterol (HDL-C_0).

15* Report numbers of outcome events or summary measures over time

(P7) After application of all exclusion criteria, 184 patients were ultimately included in the retrospective analysis as the final study cohort. The detailed patient screening and enrollment process is illustrated in Figure 1.

16 (b) Report category boundaries when continuous variables were categorized

(P4) SUA levels were defined per the Chinese Guidelines for Diagnosis and Treatment of Hyperuricemia and Gout (2023), with thresholds set at >420 μmol/L for males and >357 μmol/L for females.

17 Report other analyses done—eg analyses of subgroups and interactions, and sensitivity analyses

(P11) Comparison by Δ_{1m}-eGFR Levels

Using the median Δ_{1m}-eGFR (4.44 mL/min/1.73 m²) as the cutoff value, the group with Δ_{1m}-eGFR < 4.44 had a mean decrease in Δ_{1m}-SUA of 10.48 μmol/L, while the group with Δ_{1m}-

eGFR ≥ 4.44 had an average increase of 48.52 μmol/L ($p < 0.001$; Figure 3G).

(P12) Comparison by Weight Loss Rate

Using the median Δ_{1m}-BMI (4.25 kg/m²) as the cutoff value, the slow weight loss group (Δ_{1m}-BMI < 4.25 kg/m²) had a mean decrease in Δ_{1m}-SUA of 6.76 μmol/L, whereas the rapid weight loss group (Δ_{1m}-BMI ≥ 4.25 kg/m²) exhibited a mean increase of 44.25 μmol/L ($p < 0.001$; Figure 3H).

Discussion

18 Summarise key results with reference to study objectives

(P18) While WC serves as a classic marker for evaluating visceral fat, theoretically, Δ1m-WC may modulate SUA levels via improvements in insulin resistance[20]—a property that could render it more clinically meaningful than weight metrics like Δ_{1m}-BMI or TWL_1. However, our findings demonstrate that after adjusting for TWL_1, the independent effect of Δ_{1m}-WC was effectively attenuated, with the previously significant mediating effect abating accordingly. Our results are consistent with those reported by Kankaya et al.[21]—namely, higher BMI_0 correlates with greater TWL_1. Additionally, the present study reveals that BMI_0 exerts an effect on Δ_{1m}-SUA by mediating Δ_{1m}-BMI. This underscores that in the early postoperative management of SUA, weight loss carries greater clinical relevance than isolated changes in waist circumference and should be prioritized during the development of clinical intervention strategies.

(P21~22) These findings provide a framework for stratified management: For patients with baseline renal impairment (eGFR: 60–89 mL/min/1.73m²), prioritization of etiological interventions (e.g., diabetes control, improvement of renal perfusion) is essential to optimize

renal function preoperatively, thereby preventing postoperative deterioration that could adversely affect SUA; For patients with baseline renal hyperfiltration (eGFR > 125 mL/min/1.73m²), postoperative eGFR decline reduces renal SUA clearance. Clinicians should closely monitor dynamic changes in eGFR and SUA in this subgroup and initiate targeted interventions in a timely manner.

19 Discuss limitations of the study, taking into account sources of potential bias or imprecision. Discuss both direction and magnitude of any potential bias

(P20) Regrettably, the absence of subject-specific genotypic data in this study precludes the ability to provide novel mechanistic insights, which constitutes one limitation of the current research.

(P21~22) A major limitation of the present study is the lack of systematic collection of detailed dietary information. All patients were advised to consume 60–80 g of whey protein daily for 6 months postoperatively, as this low-purine, high-quality protein exerts minimal potential impact on SUA levels. However, due to inadequate documentation of patients' overall dietary patterns—including the potential intake of high-purine animal proteins commonly observed in postoperative metabolic and bariatric diets—we cannot rule out the potential impact of dietary purine load (independent of the study-recommended whey protein) on the 44.25 μmol/L elevation in Δ1m-SUA observed in the rapid weight loss group. Additionally, it remains unclear whether the divergent trends—Δ_{6m}-TP elevation in the Postop-6m EUA group versus a decreasing trend in the Postop-6m NUA group—are associated with postoperative dietary patterns.

Sex hormones play a critical role in regulating SUA metabolic homeostasis: Estrogen

reduces UA reabsorption by downregulating SLC2A9 expression and modulates the cellular localization and stability of ABCG2 via estrogen receptor signaling pathways, thereby promoting UA excretion—a key mechanism underlying the lower baseline SUA levels in women[32]. Post-LSG reduction in adipose tissue induces alterations in sex hormone levels: increased postoperative testosterone secretion in males selectively upregulates Smct1, enhancing the driving force for renal UA reabsorption[33,34]. This may represent a potential mechanism explaining the more pronounced postoperative SUA elevation in the male NUA group. However, sex hormone-related assays were not included in the study's assessment panel, precluding the integration of sex hormone levels and their regulatory effects on UA metabolism into statistical analyses. This constitutes another major limitation of the present research.

20 Give a cautious overall interpretation of results considering objectives, limitations, multiplicity of analyses, results from similar studies, and other relevant evidence

(P18) While WC serves as a classic marker for evaluating visceral fat, theoretically, Δ1m-WC may modulate SUA levels via improvements in insulin resistance[20]—a property that could render it more clinically meaningful than weight metrics like Δ_{1m}-BMI or TWL_1. However, our findings demonstrate that after adjusting for TWL_1, the independent effect of Δ_{1m}-WC was effectively attenuated, with the previously significant mediating effect abating accordingly. Our results are consistent with those reported by Kankaya et al.[21]—namely, higher BMI_0 correlates with greater TWL_1. Additionally, the present study reveals that BMI_0 exerts an effect on Δ1m-SUA by mediating Δ_{1m}-BMI. This underscores that in the early postoperative management of SUA, weight loss carries greater clinical relevance than isolated changes in waist circumference and should be prioritized during the development of clinical intervention

strategies.

Per IFSO guidelines[22], patients who have undergone MBS should adhere to a high-protein, low-carbohydrate, and low-fat dietary pattern. With this dietary regimen, the body undergoes rapid lipolysis during the early postoperative period—a process further augmented by minimal carbohydrate intake. When acetyl-CoA generated from fat breakdown surpasses the metabolic capacity of the tricarboxylic acid cycle, ketone body production increases substantially. This, in turn, inhibits renal SUA excretion via multiple molecular mechanisms, ultimately resulting in elevated SUA levels.

(P20) SLC2A9 variants alter UA transmembrane transport efficiency: when SLC2A9 variants enhancing reabsorption coexist with ABCG2 defects, the concomitant "reduced excretion and increased reabsorption" leads to marked SUA elevation. Conversely, SLC2A9 variants impairing function partially offset ABCG2 defects, yielding a blunted SUA response. URAT1 variants modulate renal reabsorption efficiency by altering protein affinity for UA, acting synergistically with ABCG2 and SLC2A9 variants to further amplify interindividual variability in SUA responses. Thus, the heterogeneous changes (elevation or reduction) in SUA levels after LSG in the present study may be partially attributed to the aforementioned genotypic differences[27,28].

(P22) Sex hormones play a critical role in regulating SUA metabolic homeostasis: Estrogen reduces UA reabsorption by downregulating SLC2A9 expression and modulates the cellular localization and stability of ABCG2 via estrogen receptor signaling pathways, thereby promoting UA excretion—a key mechanism underlying the lower baseline SUA levels in women[32]. Post-LSG reduction in adipose tissue induces alterations in sex hormone levels:

increased postoperative testosterone secretion in males selectively upregulates Smct1, enhancing the driving force for renal UA reabsorption[33,34]. This may represent a potential mechanism explaining the more pronounced postoperative SUA elevation in the male NUA group.

21 Discuss the generalisability (external validity) of the study results

(P22) Additionally, the single-center retrospective observational design, focus on a single surgical modality, modest sample size of 184 patients, and inherent heterogeneity between preoperative groups may restrict the generalizability of the findings. Furthermore, as a retrospective exploratory analysis without statistical correction for multiple comparisons, this study confers an elevated risk of Type I errors.

22 Give the source of funding and the role of the funders for the present study and, if applicable, for the original study on which the present article is based

(P25~26) Ethics Approval and Consent to Participate

This study was conducted in accordance with the principles of the Declaration of Helsinki, and this study was approved by the Ethics Committee of The First Hospital Affiliated to Hebei Medical University (No. 2025-108). The requirement for informed consent was waived by the Ethics Committee of The First Hospital Affiliated to Hebei Medical University because of the retrospective nature of the study.

Clinical trial number

Not applicable.

Consent for publication

Not applicable.

Availability of data and materials

The datasets used and analysed during the current study are available from the corresponding author on reasonable request.

Competing Interests

The authors declare that they have no competing interests.

Funding

Not applicable.

Authors' contributions

This perspective was initially proposed by W and G. F further developed the theory and performed the corresponding calculations. Z validated the analytical methods. All authors discussed the results and contributed to the final manuscript.

Acknowledgements

Not applicable.
